# Supplementary material for: Development of SSR Markers and Evaluation of Genetic Diversity of Endangered Plant Saussurea involucrata
Source: Biomolecules. 2024 Aug 15;14(8):1010. doi: 10.3390/biom14081010 (PMC11353235; doi:10.3390/biom14081010)
Supplement: Supplementary file 1 [file biomolecules-14-01010-s001.zip › 8.14 Supplement Figure S1.pdf]

The best grouping number was 4 based on the DK estimation

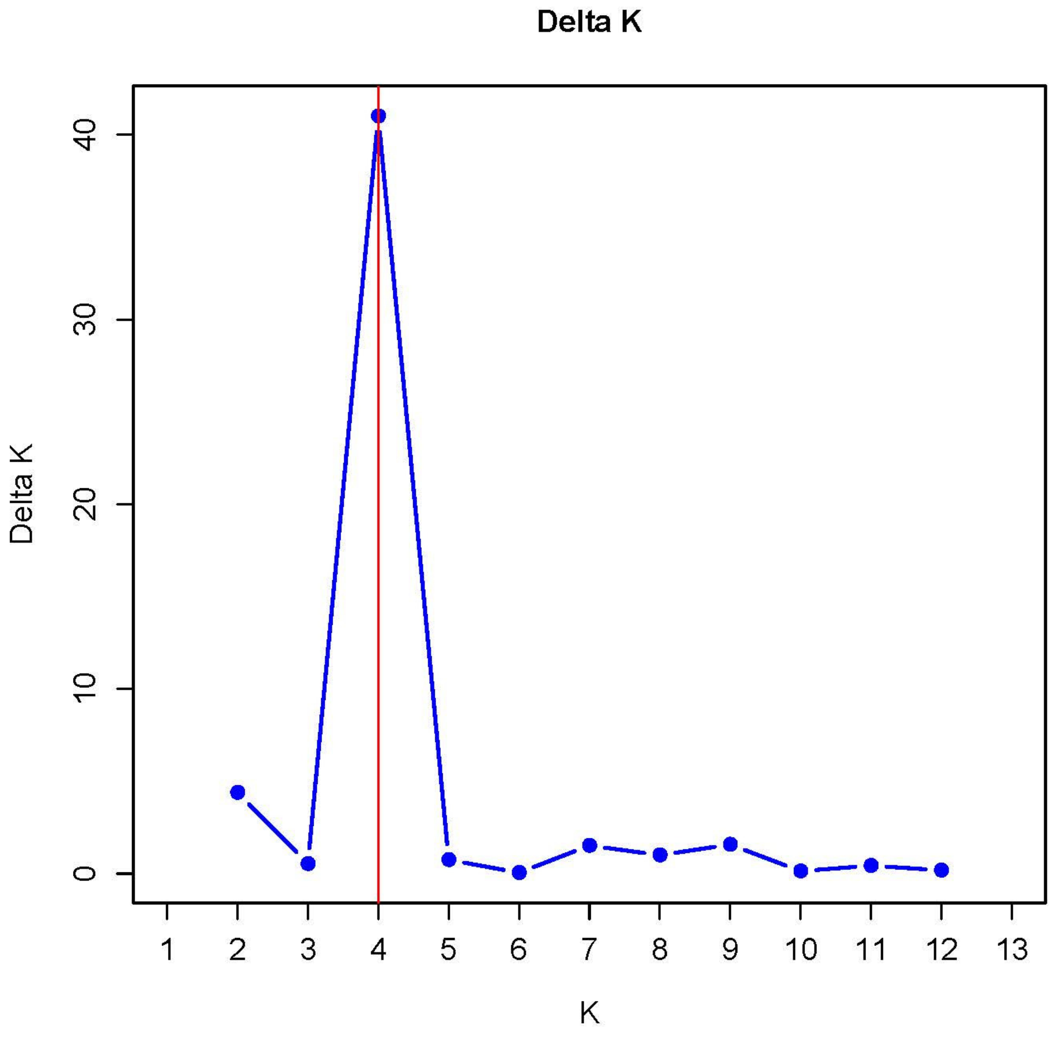

The genetic structure of *Saussurea involucrata* was inferred by Bayesian clustering of SSR data. The optimal and sub-optimal grouping number based on DK estimation is 4. 112 individuals were assigned to genetically distinguishable groups. Each population is represented by a vertical bar, colored according to the specified group.

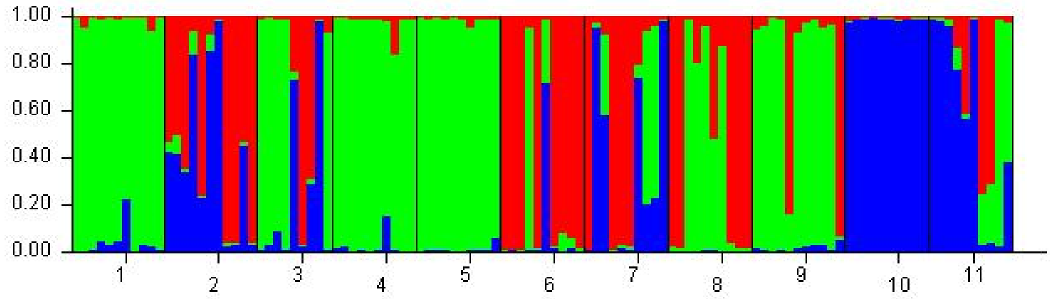

K=3

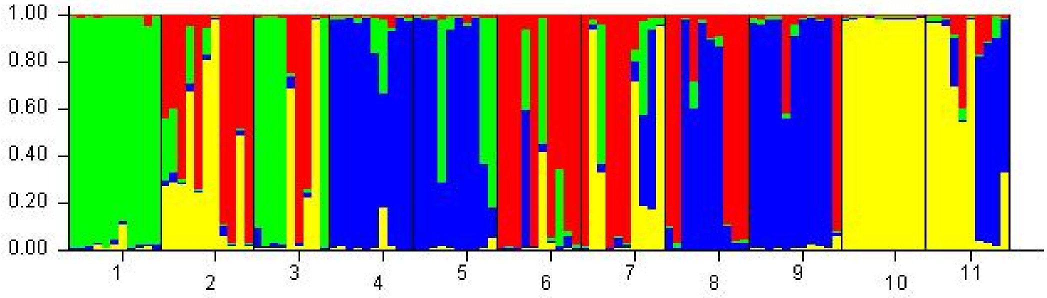

K=4

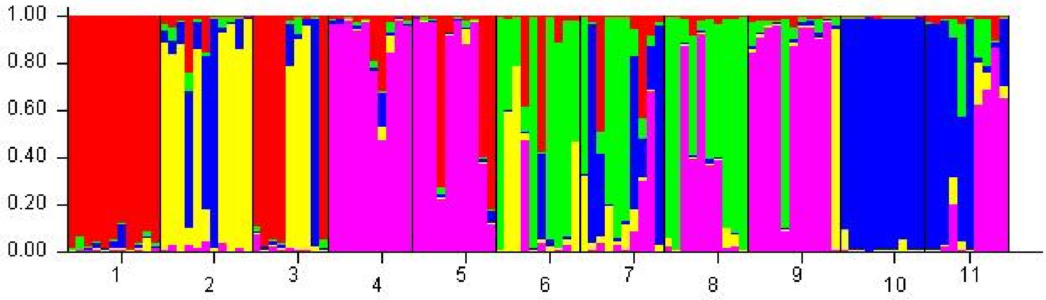

K=5

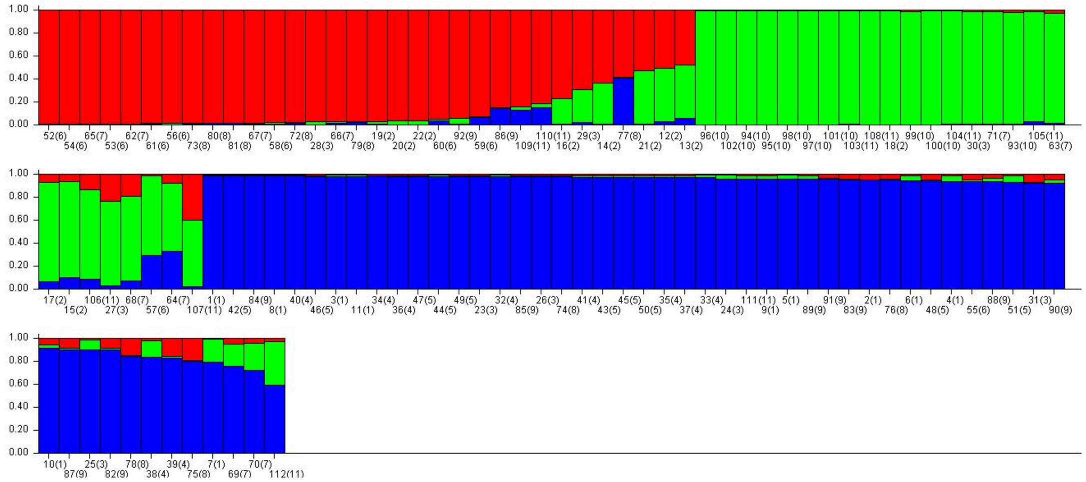
1.00
0.80
0.60
0.40
0.20
0.00

K=3

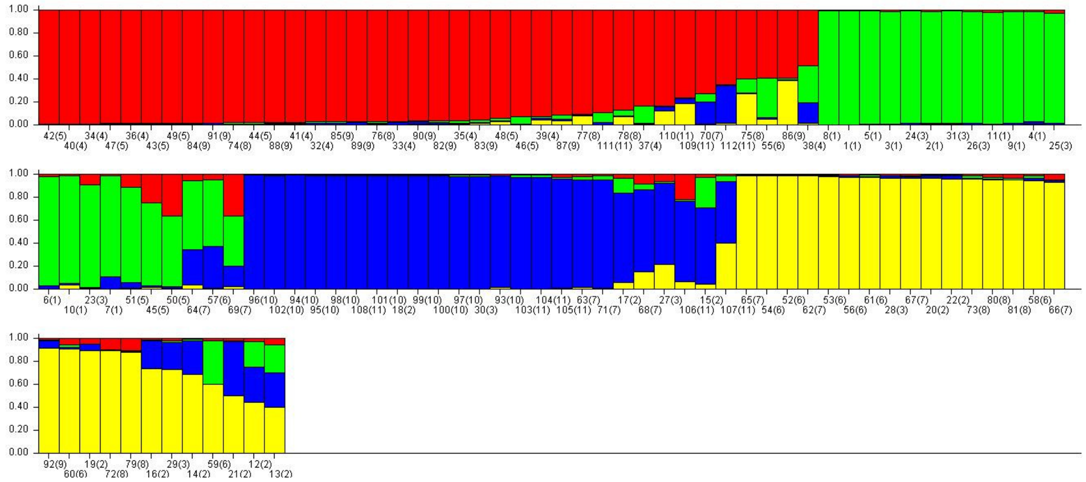
1.00
0.80
0.60
0.40
0.20
0.00

K=4

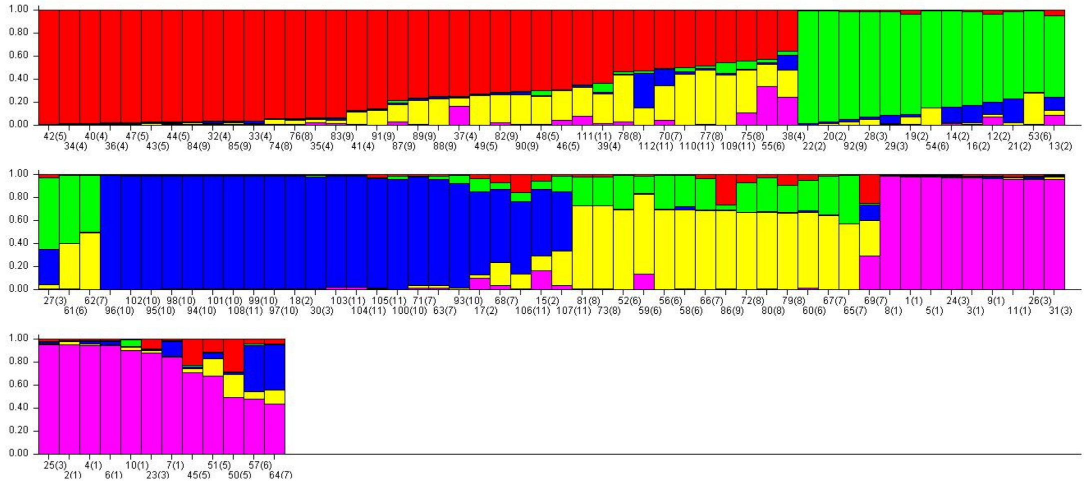
1.00
0.80
0.60
0.40
0.20
0.00

K=5
